# Supplementary material for: Implementation and Baseline Evaluation of an Evidence-Based Group Antenatal Care Program in Two Nigerian States
Source: Int J Environ Res Public Health. 2024 Oct 31;21(11):1461. doi: 10.3390/ijerph21111461 (PMC11593366; doi:10.3390/ijerph21111461)
Supplement: Supplementary file 1 [file ijerph-21-01461-s001.zip › gANC Manuscript_Supplementary Figures_09122024.pdf]

Supplementary Figures

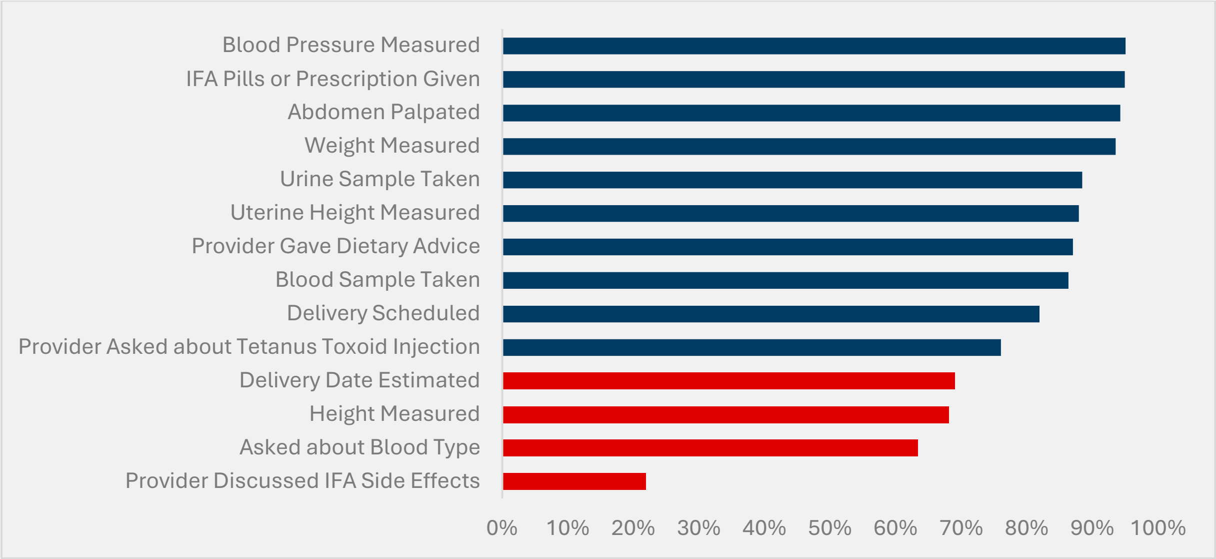

Figure S1: ANC Services Received at Most Recent Visit in Kaduna

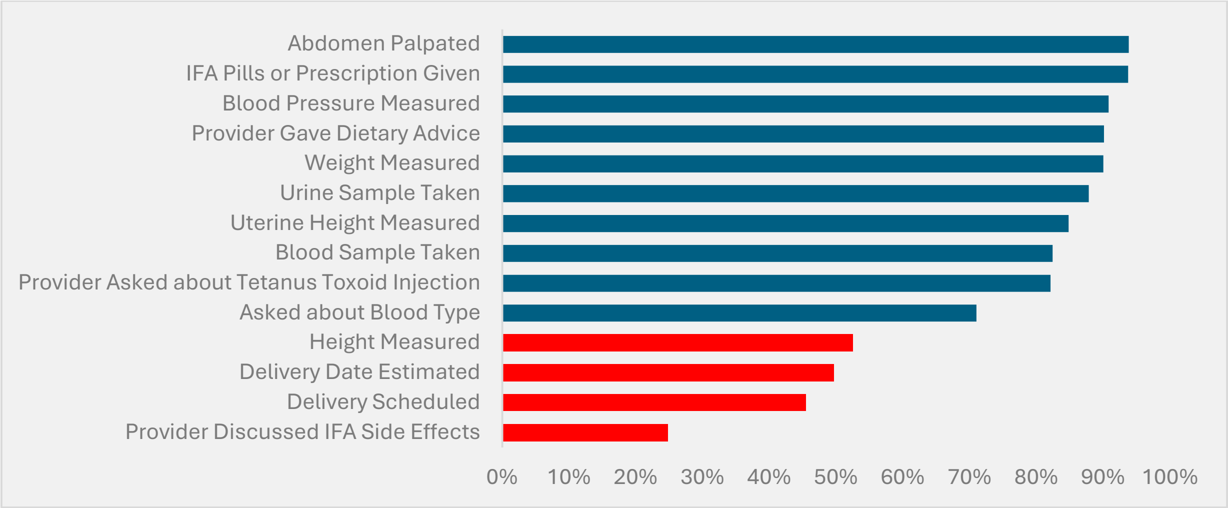

Figure S2: ANC Services Received at Most Recent Visit in Kano

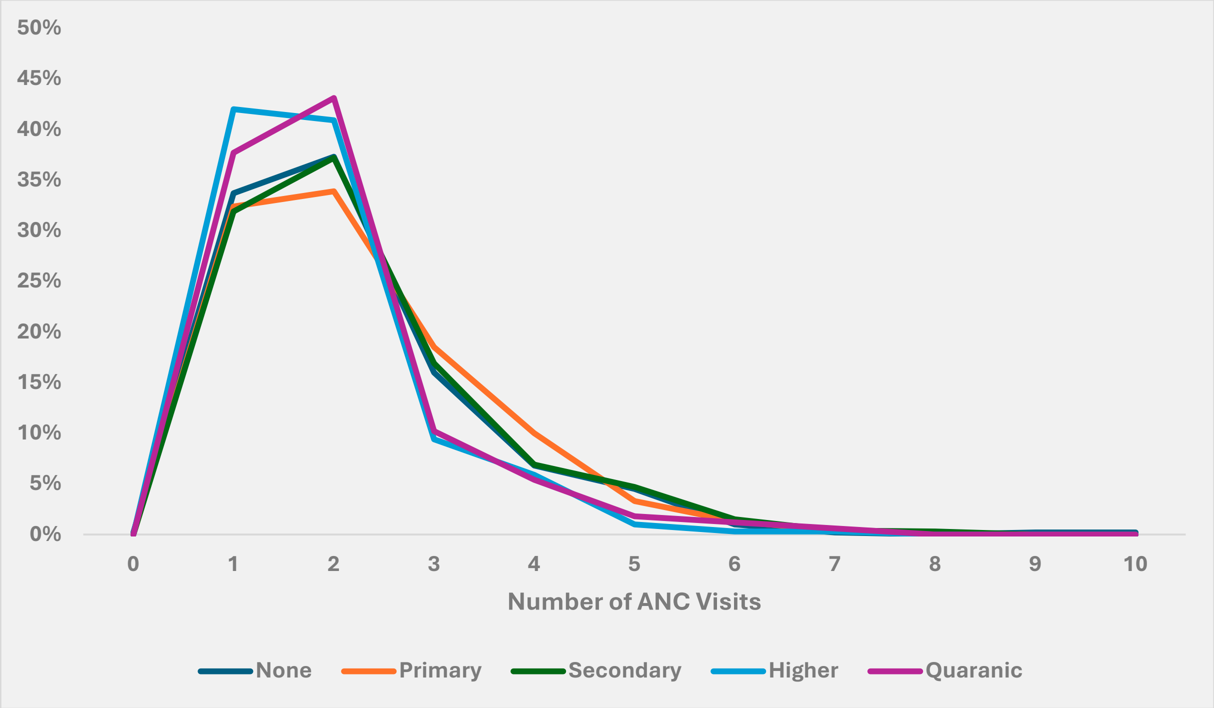

Figure S3: ANC Visits by Education Level
