# Supplementary material for: Implementation and Baseline Evaluation of an Evidence-Based Group Antenatal Care Program in Two Nigerian States
Source: Int J Environ Res Public Health. 2024 Oct 31;21(11):1461. doi: 10.3390/ijerph21111461 (PMC11593366; doi:10.3390/ijerph21111461)
Supplement: Supplementary file 1 [file ijerph-21-01461-s001.zip › gANC Manuscript_Supplementary Tables_09122024.pdf]

Supplementary Tables

Table S1: Responses to Delivery Planning Questions among Participants Who Had Identified a Facility for Delivery

Questions & Response Categories	Kaduna (n=1111)	Kano (n=638)	Total (n=1749)
Have you made any transport plan to a skilled care site for emergency?			
Yes	71.6%	77.6%	73.8%
No	27.9%	22.3%	25.8%
Don’t know	0.5%	0.2%	0.3%
Have you made cash plans for emergency care?			
Yes	68.8%	79.2%	72.6%
No	30.5%	20.7%	26.9%
Don’t know	0.7%	0.2%	0.5%
Have you made arrangement for a companion to the emergency care?			
Yes	90.1%	97.5%	92.8%
No	9.5%	2.5%	6.9%
Don’t know	0.5%	0.0%	0.3%
Are you planning to stay in this area for the next 1 and a half years and continue your antenatal care at this facility?			
Yes	94.7%	95.5%	95.0%
No	3.4%	3.3%	3.4%
Don’t know	1.9%	1.3%	1.7%
Are you planning to be away from home (near this facility) for more than 4 weeks in a row at any			

time during your pregnancy or more than 6 months in a row in the year after you have your baby?			
Yes	10.1%	15.2%	11.9%
No	87.2%	83.4%	85.8%
Don't know	2.7%	1.4%	2.2%
How long does it take you to get to the healthcare facility using your usual mode of transportation?			
<30 minutes	63.5%	67.9%	65.1%
30-59 minutes	30.1%	27.6%	29.2%
≥60 minutes	6.4%	4.5%	5.7%

Table S2: Perception of Pregnancy and Family Planning by Education Level

	None (n=576)	Primary (n=519)	Secondary (n=921)	Higher (n=286)	Quranic (n=167)	Total (n=2,469)
Do you think there are advantages to spacing pregnancies apart?						
Yes	74.3%	87.5%	92.4%	99.7%	83.2%	87.4%
No	13.4%	7.1%	4.3%	0.0%	6.0%	6.6%
Don't Know	2.3%	5.4%	3.3%	0.3%	10.8%	6.0%
Were you aware of any danger/warning signs during pregnancy?						
Yes	83.2%	90.0%	93.5%	98.3%	94.6%	91.0%
No	12.7%	7.9%	5.6%	1.4%	4.2%	7.2%
Don't Know	4.2%	2.1%	0.9%	0.3%	1.2%	1.9%
Have you made any transport plan to a skilled care site for emergency?						
Yes	69.1%	71.8%	73.4%	80.3%	77.7%	73.8%
No	30.9%	27.7%	26.4%	19.3%	21.4%	25.8%
Don't Know	0.0%	0.6%	0.3%	0.4%	0.9%	0.3%

How many ANC visits have you had?						
0	0.2%	0.0%	0.0%	0.0%	0.0%	0.0%
1	33.7%	32.4%	31.9%	42.0%	37.7%	34.0%
2	37.3%	33.9%	37.2%	40.9%	43.1%	37.4%
3	16.0%	18.5%	16.9%	9.4%	10.2%	15.7%
4	6.8%	10.0%	6.9%	5.9%	5.4%	7.3%
5	4.5%	3.3%	4.7%	1.0%	1.8%	3.7%
6	1.0%	1.3%	1.5%	0.3%	1.2%	1.2%
7	0.2%	0.4%	0.4%	0.3%	0.6%	0.4%
8	0.0%	0.2%	0.3%	0.0%	0.0%	0.2%
9	0.2%	0.0%	0.0%	0.0%	0.0%	0.0%
10	0.2%	0.0%	0.0%	0.0%	0.0%	0.0%
Are you aware of any danger/warning signs after a baby is born?						
Yes	76.9%	83.6%	83.7%	91.3%	85.6%	83.1%
No	23.1%	16.4%	16.3%	8.7%	14.4%	16.9%
Where do you intend to deliver this pregnancy?						
At home	54.9%	32.6%	12.8%	2.1%	37.7%	27.2%
At a healthcare facility	35.6%	60.5%	84.3%	97.6%	55.1%	67.5%
Undecided	9.5%	6.9%	2.8%	0.3%	7.2%	5.3%
Don’t Know	0.0%	0.0%	0.1%	0.0%	0.0%	0.0%
Did you attend any postnatal care appointments after giving birth? (n=1,950)						
Yes	75.8%	84.8%	90.6%	90.0%	87.9%	85.2%
No	23.8%	14.8%	8.5%	8.5%	11.4%	14.1%
Don’t Know	0.4%	0.4%	0.9%	1.4%	0.7%	0.7%
Could you ask your husband/partner to use a condom if you wanted him to?						
Yes	31.8%	47.8%	57.4%	67.5%	37.1%	49.2%
No	59.0%	42.2%	36.5%	25.9%	43.7%	42.2%
Depends / Don’t Know	9.2%	10.0%	6.1%	6.6%	19.2%	8.6%
